# Differential expression of estrogen receptor–α on follicular dendritic cells from patients with grade 1‐2 and grade 3 follicular lymphoma

**DOI:** 10.1002/hon.2577

**Published:** 2019-03-20

**Authors:** Rintaro Ohe, Hong‐Xue Meng, Naing Ye Aung, Akane Yamada, Takanobu Kabasawa, Aya Utsunomiya, Nobuyuki Tamazawa, Yuka Tamura, Takumi Kitaoka, Toshio Hashimoto, Kenichi Shibata, Tomomi Toubai, Wataru Kimura, Kenichi Ishizawa, Mitsunori Yamakawa

**Affiliations:** ^1^ Department of Pathological Diagnostics Yamagata University Faculty of Medicine Yamagata Japan; ^2^ Department of Pathology Harbin Medical University Cancer Hospital Harbin China; ^3^ Department of Neurology, Hematology, Metabolism, Endocrinology and Diabetology Yamagata University Faculty of Medicine Yamagata Japan; ^4^ Department of Surgery Yonezawa City Hospital Yonezawa Japan; ^5^ Department of Gastroenterological, General, Breast and Thyroid Surgery Yamagata University Faculty of Medicine Yamagata Japan

**Keywords:** CD23, estrogen receptor alpha, follicular dendritic cell, follicular lymphoma

## Abstract

Hormone therapy has been used for patients with estrogen receptor alpha (ERα)–positive breast cancers. Recently, some studies reported the expression of ERα on neoplastic cells from B‐cell lymphomas. However, there has been only one report of ERα expression on the follicular dendritic cells (FDCs) that structurally and functionally support the microenvironment of follicular lymphomas (FLs). The objective of this study was to investigate the frequency of ERα expression on FDCs in nonneoplastic reactive lymphoid tissues and to compare the frequency of ERα expression on FDCs in the axillary lymph nodes between patients with and without antiestrogen therapy and among patients with grades 1‐3 of FL. Reverse transcription–polymerase chain reaction was performed to detect ERα mRNA in FL. In nonneoplastic germinal centers (GCs) from patients with tonsillitis or reactive lymphadenitis, ERα was expressed in the light zone. ERα‐positive cells strongly correlated with the width of GCs (*r*_s_ = 0.81, *P* < 0.01) and the CD21‐positive (*r*_s_ = 0.69, *P* < 0.01) and CD23‐positive (*r*_s_ = 0.83, *P* < 0.01) FDC meshwork. The axillary lymph nodes had fewer ERα‐positive cells, smaller GCs, and a looser CD21‐ and CD23‐positive FDC meshwork with hormone therapy than without hormone therapy (*P* < 0.01). Neoplastic follicles of G1‐2 FL had more ERα‐positive cells and a larger CD23^+^ FDC meshwork than those of G3 FL (*P* < 0.01). ERα mRNA was detected in both G1‐2 FL and G3 FL by reverse transcription–polymerase chain reaction. In conclusion, these results suggested that antiestrogen hormone therapy may decrease the number of ERα‐positive FDCs and that the responses mediated by the estrogen‐ERα interaction on FDCs may differ between G1‐2 FL and G3 FL.

## 1 INTRODUCTION

Antiestrogen hormone therapies consisting of tamoxifen and fulvestrant have been used as standard treatments for patients with breast cancers expressing estrogen receptor (ER), progesterone receptor (PgR), or both.^1^ A few studies identified possible differences in ERα, ERβ, and glucocorticoid receptor expression related to B‐cell malignancies, such as chronic lymphocytic leukemia, Hodgkin lymphoma, Burkitt lymphoma, mantle cell lymphoma, and lymphoma cell lines.^2^, ^3^ Among the lymphoid stromal cells, CD21^+^CD23^+^ follicular dendritic cells (FDCs) in nonneoplastic lymph nodes (LNs) express ERα, and tamoxifen treatment affects the germinal centers (GCs) and induces the proliferation of ERα^+^ FDCs.^4^

Follicular lymphoma (FL) is a GC‐derived lymphoma^5^ that frequently follows an indolent clinical course.^6^, ^7^ Although FL is responsive to initial chemotherapy, late relapses are common.^8^ In FL, the FDCs form a meshwork in extended neoplastic follicles and may correlate with the production of some collagen‐modifying enzymes, such as lysyl hydroxylase 3, protein disulfide isomerase, and prolyl 4‐hydroxylase.^5^ FDCs support angiogenesis, cellular adhesion and migration, and the survival of FL cells, which results in the protection of lymphoma cells against apoptosis and the augmentation of multidrug resistance effects,^7^, ^9^ although it is generally known that t(14;18) protects against apoptosis of lymphoma cells themselves in 85% to 90% of FLs.^10^ The growth and survival of an FL cell line (FLK‐1) was also found to be dependent on an FDC‐like cell line (HK).^11^

To the best of our knowledge, it remains unknown whether FDCs in GC‐forming lymphomas express ERα. The objective of this study was to investigate the frequency of ERα expression on FDCs in nonneoplastic tonsillar and LN tissues and to compare the frequency of ERα expression on FDCs in axillary LNs between cases with and without antiestrogen therapy and among grades 1‐3 FL.

## 2 MATERIALS AND METHODS

### 2.1 Patients and tissue specimens

Ninety‐five patients were included; tissues included 68 tissue samples from FL patients and 27 samples of nonneoplastic lymphoid tissues, including tonsil samples from patients with chronic tonsillitis (n = 5), mesenteric LN samples from colon cancer patients (n = 5), and axillary LN samples from patients with (n = 9) and without (n = 8) antiestrogen therapy. Pathological diagnoses were determined at Yamagata University Hospital and Yonezawa City Hospital in Japan and Harbin Medical University Cancer Hospital in China between 1997 and 2018. Breast cancer patients received a luteinizing hormone–releasing hormone (leuprorelin acetate or goserelin acetate), an aromatase inhibitor (anastrozole, exemestane, or letrozole), a selective ER modulator (tamoxifen citrate or toremifene citrate), and/or a selective ER downregulator (fulvestrant) as hormone therapy. Neoplastic FL tissue specimens were obtained from patients who underwent biopsy for pathological diagnosis. The cases of FL were classified as grade 1‐2 (G1‐2, n = 32) or grade 3 (G3A, n = 22; G3B, n = 14) in accordance with the WHO Classification revised fourth edition.^10^ G1 and G2 FLs were combined because of a lack of clinically significant differences between the classifications.^10^ The histological pattern of these cases was classified as follicular (n = 50), follicular and diffuse (n = 8), focal follicular/predominantly diffuse (n = 7), and diffuse (n = 3) in accordance with the WHO Classification revised fourth edition.^10^ Tissues were fixed in 10% neutral‐buffered formalin for 6 to 12 hours at room temperature, embedded in paraffin, and used for hematoxylin‐eosin staining, immunohistochemistry, and reverse transcription–polymerase chain reaction (RT‐PCR). This study was approved by the Research Ethics Committee of Yamagata University Faculty of Medicine (H29‐343) and the Research Ethics Committee of Harbin Medical University Cancer Hospital (KY2016‐25) and was performed in accordance with the Declaration of Helsinki.

### 2.2 Immunostaining

**Figure 1:**
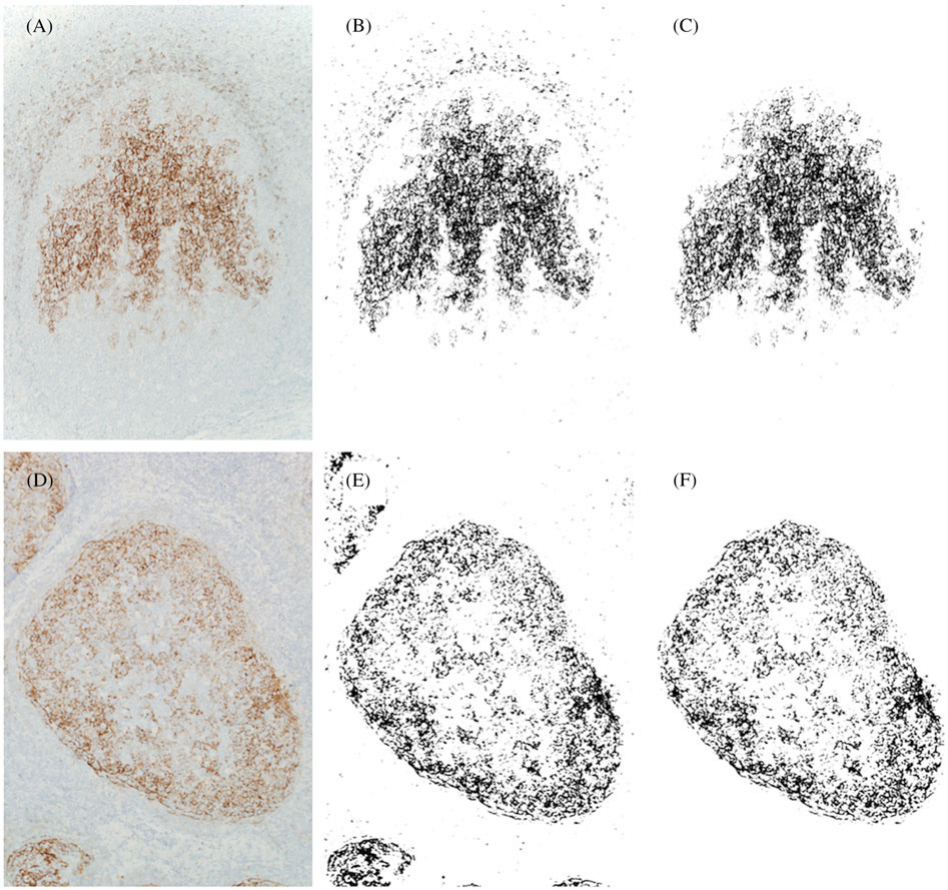
Immunohistochemical and binary images of CD23 immunostaining. An image of CD23 immunostaining in a tonsillar lymphoid follicle was captured A, and converted to a binary image by ImageJ B. The CD23^+^ follicular dendritic cell meshwork (black area) was extracted from the binary image, and the immunostained area was calculated by ImageJ C. The same method was applied to follicular lymphomas D‐F

Immunostaining was performed as previously described^12^ and used antibodies specific for ERα (EP1; rabbit IgG, DAKO, Agilent Technologies, Santa Clara, California), phosphorylated ERα [pER (ser118)] (16J4; mouse IgG2b, Cell Signaling Technology, Danvers, Massachusetts), PgR (PgR 636; mouse IgG1κ, DAKO, Agilent Technologies), CD21 (1F8; mouse IgG1κ, DAKO, Agilent Technologies), CD23 (DAK‐CD23; mouse IgG1κ, DAKO, Agilent Technologies), CD23 (SP23; rabbit IgG, Nichirei, Tokyo, Japan), CD35 (RLB25; mouse IgG2b, Novocastra, Leica Biosystems, Nussloch, Germany), Clusterin (7D1; mouse IgG1κ, Novocastra, Leica Biosystems), and Podoplanin (D2‐40; mouse IgG1κ, DAKO, Agilent Technologies). Single immunostaining for ERα, PgR, CD21, and CD23 was performed using an Autostainer Link 48 system (Agilent Technologies), while immunostaining for pER was performed manually. The estimation of the reactivity is described below. Cells positive for ERα and PgR were counted in five nonneoplastic/neoplastic follicles for each case. Cells positive for pER (ser118) were counted in five neoplastic follicles for each case. In FL cases with diffuse proliferation, positive reactions were counted in five high‐power fields. GCs were identified in two areas: The light zone (LZ) was positive for CD23, while the dark zone (DZ) was negative for CD23.^13^ The area of the FDC meshwork expressing CD21 or CD23 was determined from photographs and estimated as the gross area by ImageJ (Figure 1).^14^, ^15^ The black and white binary images highlighted the FDC meshwork, which was calculated as the black area (μm^2^). The GC area was estimated to have a polygonal area (μm^2^). Double immunostaining was performed as previously described.^16^ A positive reaction of the first antibody (specific for ERα) was detected as a brown coloration with 3,3′‐diaminobenzidine tetrahydrochloride (Dojindo, Kumamoto, Japan), and a positive reaction of the second antibody (specific for CD23, CD35, Clusterin, or Podoplanin) was detected as a red coloration with Vulcan Fast Red (Biocare Medical, Pacheco, California). Breast and ovarian cancers were used as positive controls for ERα and pER (ser118), respectively. In contrast, FLEX Universal Negative Controls for mouse and rabbit (DAKO, Agilent Technologies) were used as negative controls for the primary antibodies.

### 2.3 Semiquantitative RT‐PCR

Paraffin‐embedded tonsil tissue sections (n = 1), which served as positive controls because ER mRNA has been detected in the GCs of LNs,^4^ and FL (G1‐2, n = 5; G3, n = 6) samples were assessed. RT‐PCR was performed as previously described.^16^ The following primer sequences were designed by PrimerBank (http://pga.mgh.harvard.edu/primerbank/): 5′‐GAAAGGTGGGATACGAAAAGACC‐3′ and 5′‐GCTGTTCTTCTTAGAGCGTTTGA‐3′ for human ERα (163 bp) and 5′‐GCACCGTCAAGGCTGAGAAC‐3′ and 5′‐TGGTGAAGACGCCAGTGGA‐3′ for human glyceraldehyde‐3‐phosphate dehydrogenase (GAPDH, 138 bp) as the internal control. cDNA was amplified by RT‐PCR in a MasterCycler 1 PCR thermal cycler (Eppendorf, Hamburg, Germany), as follows: 94°C for 10 minutes; 30 cycles of 94°C for 30 seconds, 58°C for 45 seconds, 72°C for 30 seconds and 1 cycle of 72°C for 6 minutes. The density of the bands was measured by densitometry, and the ratios of ERα to GAPDH mRNA were calculated by ImageJ.^14^, ^15^

### 2.4 Statistical analysis

The Mann‐Whitney test was performed to compare the number of ERα^+^ cells in secondary lymphoid tissues and FL tissues. Spearman rank correlation was used to correlate the number of ERα^+^ cells with the GCs, the CD21^+^ FDC meshwork, and the CD23^+^ FDC meshwork. Spearman coefficient was denoted by *r*_s_. A receiver operating characteristic (ROC) curve was constructed using JMP, version 14 (SAS Institute, Tokyo, Japan). Statistical analyses were performed using JMP. Differences for which *P* < 0.05 were considered significant in each analysis.

## 3 RESULTS

### 3.1 ERα expression on CD23^+^ FDCs in GCs from nonneoplastic lymphoid tissues

**Figure 2:**
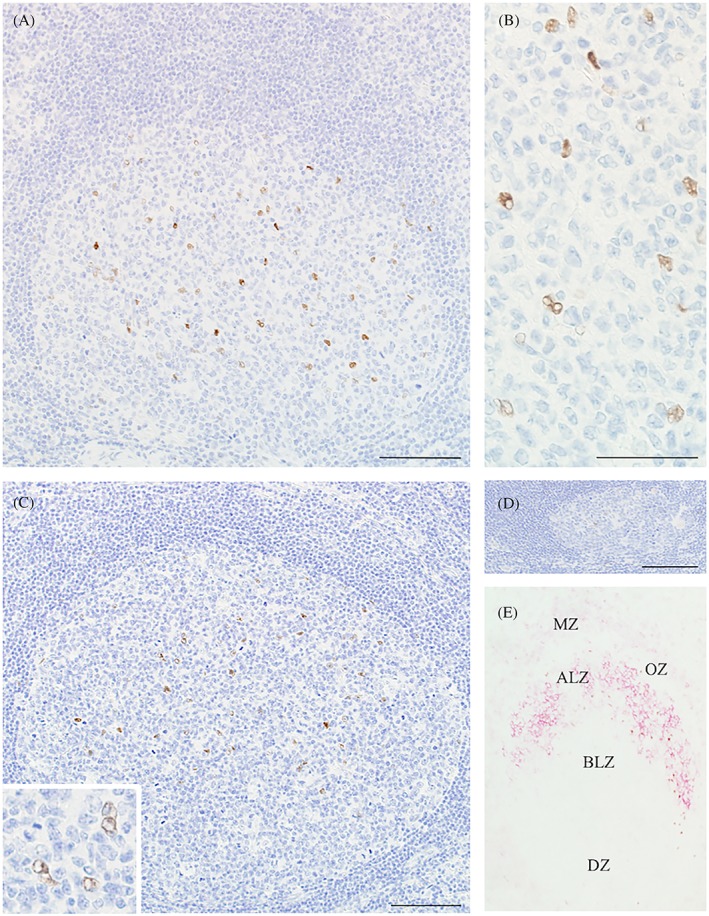
Estrogen receptor α (ERα) expression on follicular dendritic cells (FDCs) in the germinal centers (GCs) of reactive lymphoid tissues. ERα was expressed in a nonneoplastic tonsillar germinal center A. A bar, 100 μm. ERα^+^ cells appeared to have a nuclear morphology similar to that of FDCs B. A bar, 50 μm. ERα was expressed in the GC of a nonneoplastic axillary lymph node (LN) C. A bar, 100 μm. ERα^+^ cells in the axillary LN had a nuclear morphology similar to that of FDCs (C: Inset). ERα was not highly expressed in the GC of a nonneoplastic axillary LN after antiestrogen therapy D. A bar, 100 μm. Double immunostaining showed ERα expression on CD23^+^ FDCs in a nonneoplastic tonsillar GC E. CD23 (red) was strongly expressed in the apical light zone and the mantle zone of a tonsillar GC. ERα (brown) was simultaneously expressed on CD23^+^ FDCs in the ALZ. ALZ, apical light zone; BLZ, basal light zone; DZ, dark zone; MZ, mantle zone; OZ, outer zone

**Figure 3:**
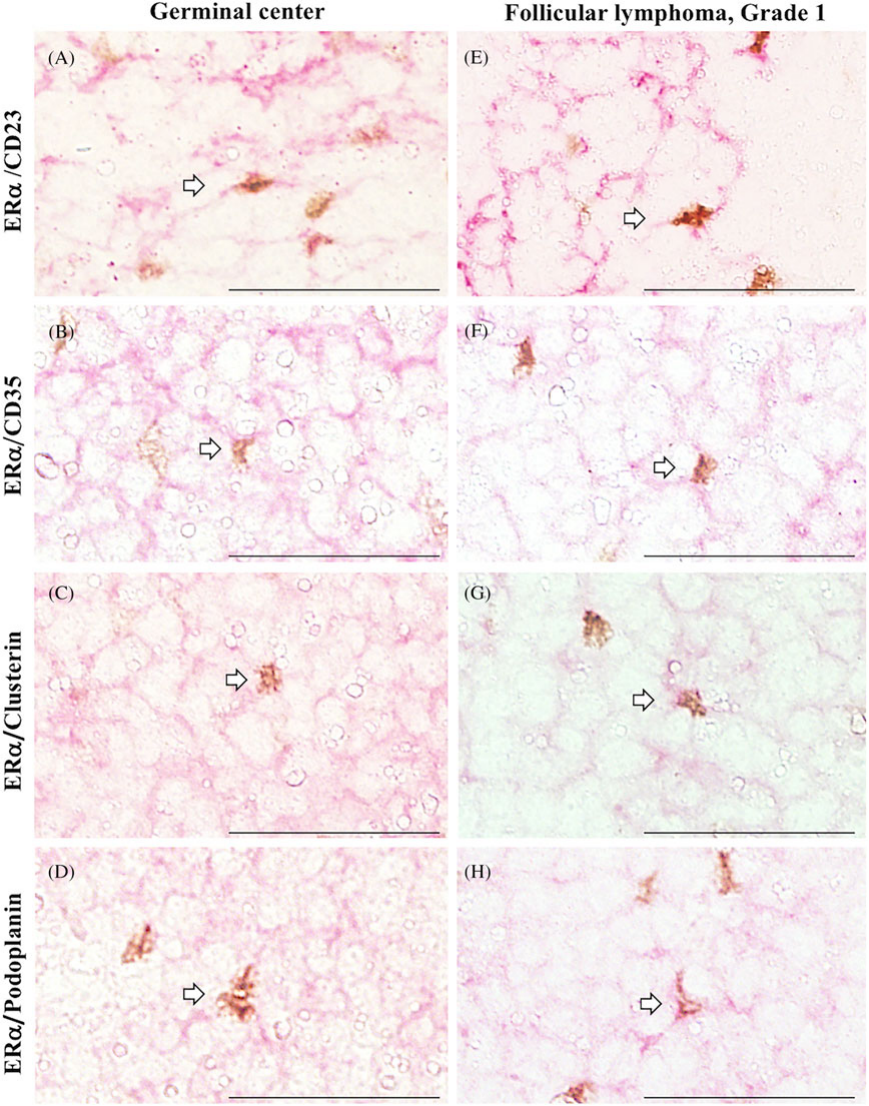
Estrogen receptor α (ERα) expression on follicular dendritic cells (FDCs) by double staining. CD23, CD35, Clusterin, and Podoplanin (red) were strongly expressed in the FDC meshwork in nonneoplastic tonsillar germinal centers A‐D, and follicular lymphoma neoplastic follicles E‐H; ERα (brown) was simultaneously expressed on FDCs A‐H. Cells double positive for CD23, CD35, Clusterin, or Podoplanin and ERα localized to both germinal centers and neoplastic follicles, as indicated by white arrows. Bars, 50 μm

The expression of ERα in nonneoplastic lymphoid tissues was evaluated by single immunostaining (Figure 2A‐D and Table 1). In accordance with the CD23^+^ FDC meshwork, ERα was expressed in the LZ of GCs in nonneoplastic lymphoid tissues (tonsil, 49.8 ± 23.7 positive cells/GC; mesenteric LN, 43.6 ± 18.9 positive cells/GC; axillary LN, 27.2 ± 18.8 positive cells/GC; and post‐hormone therapy axillary LN, 12.3 ± 11.5 positive cells/GC) but not in the DZ. ERα was expressed at lower levels (Figure 2D) in the axillary LNs after antiestrogen therapy than in other nonneoplastic lymphoid tissues (*P* < 0.01). ERα was expressed on large nuclei, which had inconspicuous nucleoli with very fine, almost vesicular chromatin. ERα^+^ cells often appeared binucleated and directly faced each other, which is compatible with the characteristic nuclear morphology of FDCs.^17^ Furthermore, by double immunostaining, we determined whether CD23^+^, CD35^+^, Clusterin^+^, or Podoplanin^+^ FDCs expressed ERα (Figures 2E and 3A‐D). The number of ERα^+^ cells in tonsillar GCs was positively correlated with the area of the CD23^+^ FDC meshwork and was not correlated with the area of GCs or the CD21^+^ FDC meshwork (Table 1). However, the number of ERα^+^ cells in GCs from the mesenteric and axillary LNs of patients treated with and without antiestrogen therapy strongly correlated with the areas of GCs and the CD21^+^ and CD23^+^ FDC meshwork. After antiestrogen therapy, the axillary LNs had fewer ERα^+^ cells and smaller areas of GCs and CD21^+^ and CD23^+^ FDC meshwork than axillary LNs from nonneoplastic lymphadenitis tissue (*P* < 0.01). After tamoxifen treatment, only one patient showed larger GCs with more abundant ERα^+^ cells and a larger CD21^+^ and CD23^+^ FDC meshwork,^4^ although two other patients treated with tamoxifen showed smaller GCs. No expression of PgR was observed on FDCs in any nonneoplastic lymphoid tissues.

**Table 1:**
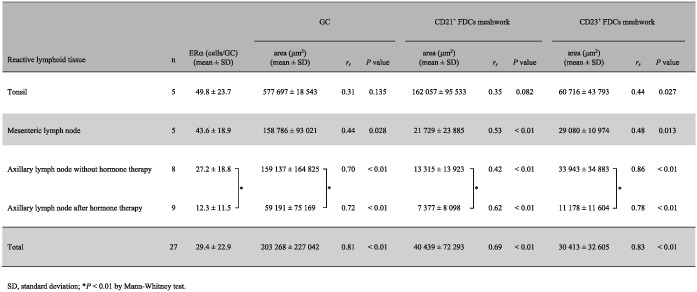
Correlation of estrogen receptor α (ERα)^+^ cells with the area of germinal left (GC) and and CD21^+^ and CD23^+^ follicular dendritic cell (FDC) meshwork in the reactive lymphoid tissues

### 3.2 Comparison of ERα expression between G1‐2 FL and G3 FL

**Figure 4:**
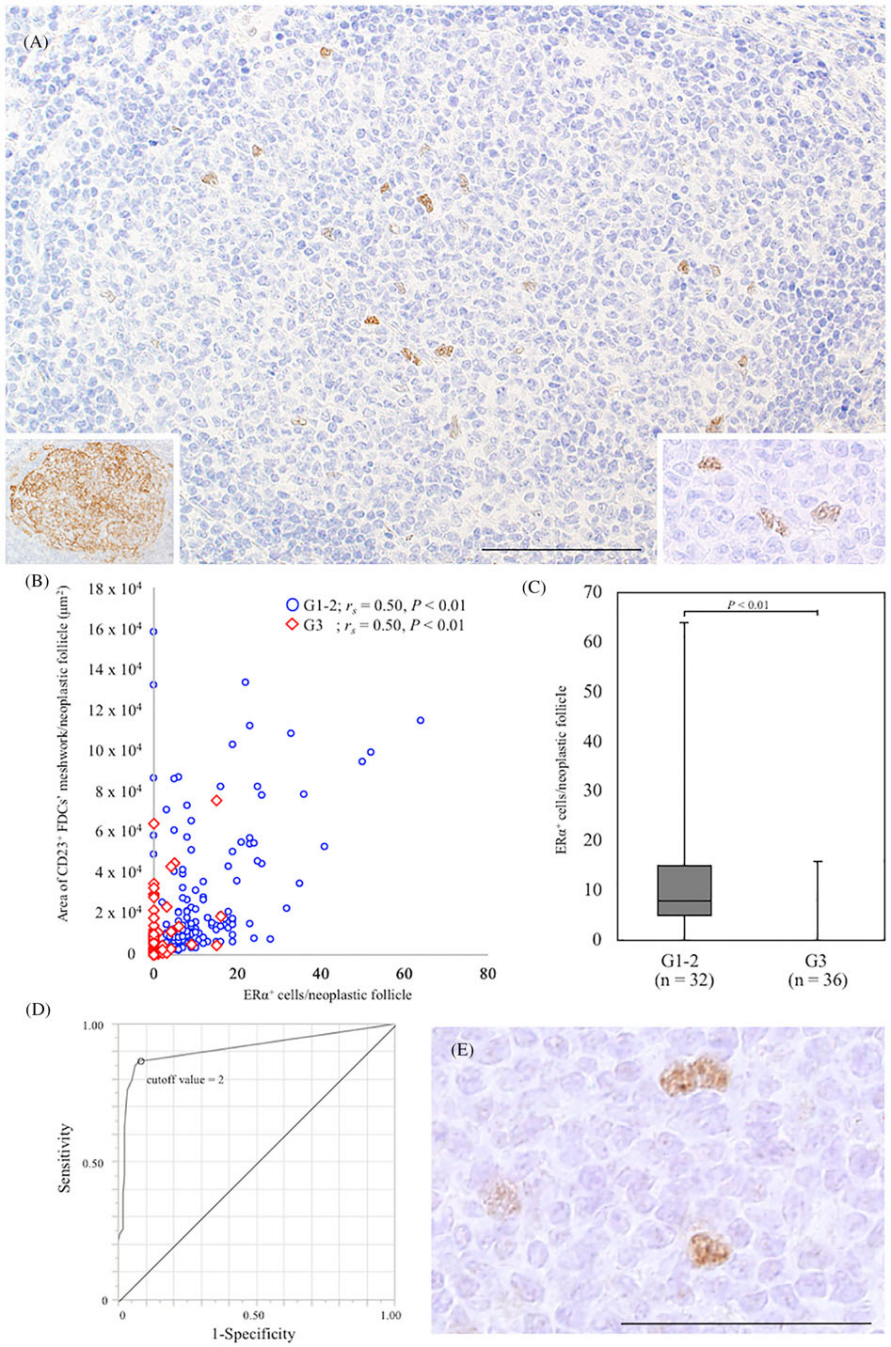
Estrogen receptor α (ERα) expression on follicular dendritic cells (FDCs) in a neoplastic follicle from follicular lymphoma (FL). ERα was expressed in a neoplastic follicle of G1 FL A. A bar, 100 μm. ERα^+^ cells in the follicle had a nuclear morphology similar to that of FDCs (A: Right inset). CD23^+^ FDC meshwork was confirmed in the same neoplastic follicle (A: Left inset). A positive correlation was observed between the number of ERα^+^ cells in a neoplastic follicle and the area of the CD23^+^ FDC meshwork (μm^2^) in G1‐2 and G3 FL B. G1‐2 FL samples had more ERα^+^ cells than G3 FL samples (*P* < 0.01) C. The number of ERα‐positive cells was counted in five neoplastic follicles in each case. The receiver operating characteristic curve of the number of ERα^+^ cells on neoplastic follicles was compared between G1‐2 FL and G3 FL. The area under the curve was 0.91. The cutoff point for the number of ERα^+^ cells per neoplastic follicle was 2 D. Phosphorylated ERα (ser118) expression on FDCs in a case of FL E. A bar, 50 μm. G1‐2, grades 1 and 2; G3, grades 3A and 3B

According to double‐immunostained FL tissues, ERα^+^ cells were simultaneously positive for CD23, CD35, Clusterin, and Podoplanin (Figure 3E‐H). ERα was expressed in the neoplastic follicles of G1‐2 FL, which was similar to its expression in GCs from nonneoplastic lymphoid follicles (Figure 4A). In G3 FL, ERα^+^ cells were absent or infrequent. The number of ERα^+^ cells in neoplastic follicles was positively correlated with the area of the CD23^+^ FDC meshwork in G1‐2 FL (*r*_s_ = 0.50, *P* < 0.01) and G3 FL (*r*_s_ = 0.50, *P* < 0.01) (Figure 4B). G1‐2 FL samples had a larger number of ERα^+^ cells in the neoplastic follicles than G3 FL samples (*P* < 0.01) (Figure 4C). ROC analysis was performed to obtain the area under the curve (AUC) and a cutoff point for the number of ERα^+^ cells per neoplastic follicle (ENF) for the classification of G1‐2 and G3 FL. The AUC of ENF was 0.91, and the cutoff point of ENF was 2 (sensitivity, 0.87; specificity, 0.92) (Figure 4D). Only two G1‐2 FL cases exhibited pER (ser118) expression on FDCs (Figure 4E). Neither ERα on lymphoma cells nor PgR on FDCs and lymphoma cells was found in FL of any grade.

### 3.3 Expression of ERα mRNA in FL by RT‐PCR

**Figure 5:**
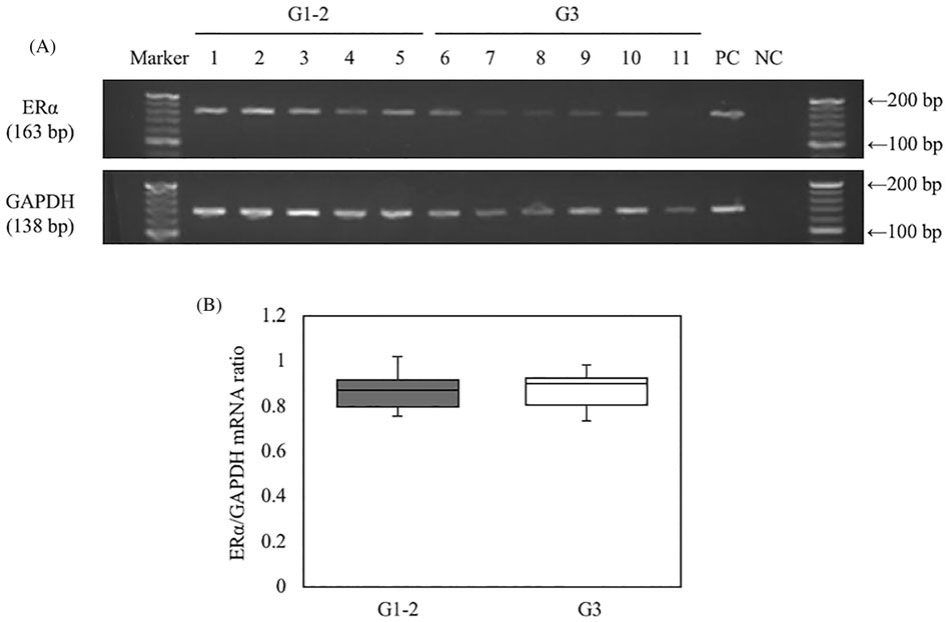
Estrogen receptor α (ERα) mRNA in follicular lymphoma (FL) as detected by reverse transcription–polymerase chain reaction (RT‐PCR). RT‐PCR was applied to measure mRNAs encoding ERα and glyceraldehyde‐3‐phosphate dehydrogenase (GAPDH) in G1‐2 and G3 FL A. Both GAPDH and ERα mRNAs were expressed in all cases of FL as well as the positive control tonsil tissue. ERα mRNA levels were determined by semiquantitative PCR and were normalized to the GAPDH mRNA levels in G1‐2 (n = 5) FL and G3 FL (n = 6) B. No significant difference was observed in the ratio of ERα to GAPDH mRNA between G1‐2 FL and G3 FL. G1‐2, grades 1 and 2; G3, grades 3A and 3B; NC, negative control; PC, positive control

RT‐PCR was performed to detect the presence of ERα mRNA in G1‐2 FL, G3 FL, and tonsil tissue. GAPDH mRNA was expressed in all cases. In addition, ERα mRNA was detected in G1‐2 FL, G3 FL, and tonsil tissue (Figure 5A). According to the semiquantitative RT‐PCR analysis by ImageJ, the ratios of ERα to GAPDH mRNA in G1‐2 FL and G3 FL did not differ significantly (Figure 5B).

## 4 DISCUSSION

We first demonstrated that ERα is expressed on CD23^+^ FDCs in both nonneoplastic lymphoid tissues, including chronic tonsillitis and reactive lymphadenitis tissues, and FL tissues. G1‐2 FL tissue had more ERα^+^ cells than G3 FL tissue, and ERα mRNA was observed in both tissue types. A previous study reported that the majority of ERα^+^ cells in GCs were CD21^+^ FDCs, but CD23^+^ FDCs were infrequent.^4^ Our results were slightly different, as they demonstrated that ERα^+^ cells localized in the LZ and mainly corresponded to the width of the CD23^+^ FDC meshwork rather than the CD21^+^ FDC meshwork, which indicates that the majority of ERα^+^ FDCs simultaneously expressed CD23. Therefore, in this study, we focused on the distribution of ERα^+^CD23^+^ FDCs in FL.

Tsunoda et al demonstrated that the FDC immunophenotypes of different grades of FL correspond to different zones of nonneoplastic GCs.^13^ For instance, the FDC immunophenotype of G1 FL resembles that of the LZ, and the FDC immunophenotype of G3 FL resembles that of the DZ.^13^ We demonstrated that ERα^+^CD23^+^ FDCs are distributed in the LZ of GCs, which may be similar to the distribution in neoplastic follicles from G1‐2 FL. No ERα^+^CD23^+^ FDCs were observed in the DZ of GCs, and few such cells were seen in neoplastic follicles from G3 FL. These results suggest that ERα^+^CD23^+^ FDCs in particular may support the neoplastic follicular microenvironment of G1‐2 FL; these FDCs could thus be a target of antiestrogen hormone therapy because, in this study, axillary LNs subjected to hormone therapy had smaller areas of GCs and FDC meshwork than axillary LNs (reactive lymphadenitis) without hormone therapy (*P* < 0.01). Thus, antiestrogen therapy for FL may decrease the areas of neoplastic follicles and the FDC meshwork and improve the disease state. A recent study supports our suggestion that a better‐developed CD21^+^ FDC meshwork is a negative prognostic factor for survival in patients with large B‐cell transformation of FL,^6^ although the extent of Ki‐M4p^+^ or CD23^+^ FDC meshwork was not associated with time to treatment failure or overall survival in FL.^18^ Tamoxifen would not be valuable for FL because pER (ser118) was expressed on FDCs in only two G1‐2 FL cases in this study, although pER (ser118) was reported as a predictive marker of a better response to tamoxifen.^19^ Additionally, the cutoff point of ENF for distinguishing G1‐2 and G3 FL was determined to be 2 in this study. Thus, if ENF is ≥2 at the time of FL diagnosis, we predict that the lesion would be G1‐2. Conversely, if ENF is 0 or only 1 at the time of FL diagnosis, we predict that the lesion would be G3. Therefore, for the classification of FL as either G1‐2 or G3, it is useful to estimate ENF in some cases when it is difficult to evaluate the absolute number of centroblasts per high‐power field.^10^

The ERα mRNA level in microdissected GCs was higher than that in the interfollicular areas in nonneoplastic LNs.^4^ In this study, ERα mRNA was detected by RT‐PCR in neoplastic FL tissue. Although a significant difference was observed in the number of ERα^+^ cells between G1‐2 and G3 FL based on the immunostaining results, no significant difference was observed in the ERα mRNA expression level according to semiquantitative RT‐PCR. This finding may have been due to low levels of ERα translation. However, these results were affected by ERα^+^ cells other than FDCs because the mRNA was extracted from not only neoplastic follicles of FL but also neighboring microenvironment. To obtain an exact quantitative comparison of ERα mRNA expression between nonneoplastic GCs and G1‐2 and G3 FL, extraction of the follicles by laser capture microdissection followed by real‐time PCR is suggested for future studies. Furthermore, in situ hybridization for ERα mRNA expression on FDC within nonneoplastic and neoplastic follicular microenvironment is also suggested for future studies.

We acknowledge several limitations of this study. First, ERα mRNA was identified only in formalin‐fixed paraffin‐embedded tissues. Second, the role of ERα on FDCs in FL is unknown.

In conclusion, we are the first to demonstrate that CD23^+^ FDCs supporting the neoplastic follicular microenvironment of G1‐2 FL simultaneously express ERα. This finding suggests a difference in the microenvironment between G1‐2 FL and G3 FL.

## CONFLICT OF INTEREST

All authors declare no conflict of interest.
